# Syntheses, structures, and magnetic properties of acetate-bridged lanthanide complexes based on a tripodal oxygen ligand

**DOI:** 10.3389/fchem.2022.1021358

**Published:** 2022-09-19

**Authors:** Yu Sheng, Yu-Jing Jiang, Zi-Hang Cheng, Ru-Chan Liu, Jing-Yuan Ge, Feng Gao

**Affiliations:** ^1^ School of Chemistry and Materials Science, Jiangsu Normal University, Xuzhou, China; ^2^ College of Chemistry and Materials Engineering, Wenzhou University, Wenzhou, China

**Keywords:** lanthanide complexes, ligand-field effect, single-molecule magnets, magnetic properties, magnetic interactions

## Abstract

Four homodinuclear lanthanide complexes, Dy_2_ (L_OEt_)_2_(OAc)_4_ (**1**), Tb_2_ (L_OEt_)_2_(OAc)_4_ (**2**), Ho_2_(L_OEt_)_2_(OAc)_4_ (**3**), and Gd_2_ (L_OEt_)_2_(OAc)_4_ (**4**), have been synthesized and characterized based on a tripodal oxygen ligand Na [(η^5^-C_5_H_5_)Co(P(O)(OC_2_H_5_)_2_)_3_] (NaL_OEt_). Structural analyses show that the acetate anions bridge two symmetry-related Ln^3+^ ions in the μ_2_:η^1^:η^1^ and μ_2_:η^1^:η^2^ coordination patterns, and each lanthanide (III) ion owns a twisted square antiprism (SAPR) conformation. Static magnetic measurements reveal the weak intramolecular ferromagnetic interaction between dysprosium (III) ions in **1** and antiferromagnetic Ln^3+^···Ln^3+^ couplings in the other three complexes. Through the analysis of the ligand-field effect and magnetic anisotropy axis orientation, the reasons for the lack of dynamic magnetic behavior in **1** were identified.

## Introduction

As novel nano-molecular magnetic materials, single-molecule magnets (SMMs), showing slow relaxation of magnetization, have attracted widespread interest in both theoretical and applied research areas because of their intriguing structures and specific physical/chemical properties (Leuenberger and Loss, 2001; Bogani and Wernsdorfer, 2008; Troiani and Affronte, 2011; Li J et al., 2021). It has been found that the introduction of paramagnetic lanthanide ions with larger magnetic anisotropy and stronger spin-orbit coupling is an effective approach to construct promising SMMs.

So far, many high-performance mononuclear lanthanide-based SMMs, also named single-ion magnets (SIMs), have been reported (Liu et al., 2018; Zhu et al., 2019; Parmar et al., 2021; Sutter et al., 2022). The studies revealed that a suitable crystal-field environment with specific symmetry around lanthanide spin centers, such as *D*_*4d*_ (Ishikawa et al., 2003; Bala et al., 2019; Zhuo et al., 2021), *D*_*4h*_ (Ding et al., 2021; Thomas-Hargreaves et al., 2021), *D*_*5h*_ (Chen et al., 2016; Ding et al., 2022; Sutter et al., 2022), *D*_*6h*_ (Canaj et al., 2019; Li et al., 2019; Zhu et al., 2021), and *C*_*∞*_ (Goodwin et al., 2017; Guo et al., 2017; Guo et al., 2018), usually leads to a remarkable single-ion magnetic anisotropy and slow magnetic relaxation behavior.

For Ln-SIMs, it is convenient to investigate the relationship between the ligand-field effect, uniaxial magnetic anisotropy, and magnetic relaxation processes. However, some factors, e.g., the higher coordination number and relatively low axisymmetric tendency of lanthanide ions and the common quantum tunneling of magnetization (QTM) effect in SIMs, still maintain a certain level of challenge for molecular design.

As an alternative, the research on polynuclear lanthanide-based SMMs, especially simple dinuclear lanthanide systems, provides a broader space for suppressing QTM and fine-tuning the dynamic magnetic behaviors by introducing intramolecular magnetic couplings (Morita et al., 2018; Gao et al., 2019; Goodwin, 2020; Gould et al., 2020; Meng et al., 2020; Li X et al., 2021; Xu et al., 2021).

**SCHEME 1:**
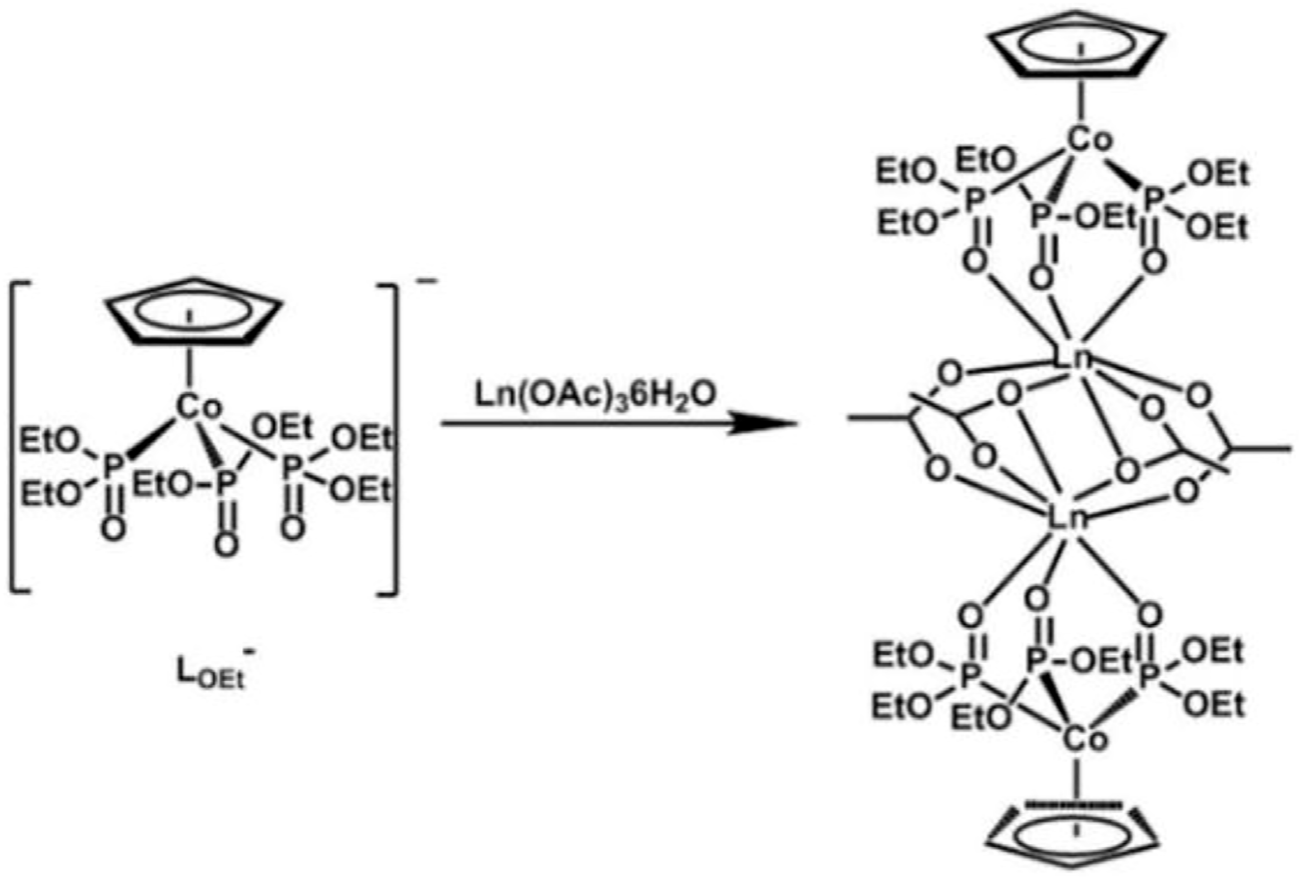
Synthetic process of target complexes Ln_2_ (L_OEt_)_2_(OAc)_4_ (Ln = Dy (**1**), Tb (**2**), Ho (**3**), and Gd (**4**)).

Many reports on dinuclear Ln-SMMs have shown that the effective regulation of local symmetry around spin carriers, magnetic anisotropy axis orientation, and the strength and nature of paramagnetic Ln^3+^···Ln^3+^ magnetic interactions through choosing appropriate ligands is still the focus of current research. Therefore, four new acetate-bridged dinuclear lanthanide complexes Dy_2_ (L_OEt_)_2_(OAc)_4_ (**1**), Tb_2_ (L_OEt_)_2_(OAc)_4_ (**2**), Ho_2_(L_OEt_)_2_(OAc)_4_ (**3**), and Gd_2_ (L_OEt_)_2_(OAc)_4_ (**4**) were designed and prepared in this study by virtue of the chelation coordination feature of a tripodal oxygen ligand Na [(η^5^-C_5_H_5_)Co(P(O)(OC_2_H_5_)_2_)_3_] (NaL_OEt_) and the variable bridge modes of acetate anion (Scheme 1). Their crystal structures and magnetic properties were also significantly investigated.

## Experimental sections

### Preparation of Dy_2_ (L_OEt_)_2_(OAc)_4_ (1)

For the preparation, 27.8 mg (0.062 mmol) Dy(OAc)_3_·6H_2_O and 35.6 mg (0.062 mmol) tripodal ligand NaL_OEt_ were dissolved in 8 ml of methanol and 5 ml of acetone. The resultant solution was treated for 10 h at 90°C. After about 6 days, suitable yellow crystals can be produced by evaporating the clear mother solution at room temperature (yield = 52%, based on NaL_OEt_). Main IR data (cm^−1^): 2977(m), 1625(s), 1447(m), 1416(m), 1142(s), 1038(s), 932(m), 832(m), 771(m), 722(m), and 583(m). Anal. Calcd for C_42_H_82_Co_2_Dy_2_O_26_P_6_ (%): C, 30.91; H, 5.07. Found: C, 31.10; H, 5.23. UV-Vis (λ_max_/nm with log (ε/dm^3^ mol^−1^ cm^−1^)): 245(4.77) and 335(3.92).

### Preparation of Tb_2_ (L_OEt_)_2_(OAc)_4_ (2)

The preparation of Tb_2_ (L_OEt_)_2_(OAc)_4_ (**2**) followed the same procedure as (**1**), using 27.5 mg (0.062 mmol) Tb(OAc)_3_·6H_2_O. Similar yellow crystals can be produced (yield = 45%, based on NaL_OEt_). Main IR data (cm^−1^): 2977(m), 1621(s), 1442(m), 1414(m), 1141(s), 1038(s), 932(m), 832(m), 771(m), 721(m), and 582(m). Anal. Calcd for C_42_H_82_Co_2_Tb_2_O_26_P_6_ (%): C, 31.05; H, 5.09. Found: C, 31.23; H, 5.26. UV-Vis (λ_max_/nm with log (ε/dm^3^ mol^−1^ cm^−1^)): 244(4.78) and 335(3.91).

### Preparation of Ho_2_(L_OEt_)_2_(OAc)_4_ (3)

The preparation of Ho_2_(L_OEt_)_2_(OAc)_4_ (**3**) followed the same procedure as (**1**), using 27.9 mg (0.062 mmol) Ho(OAc)_3_·6H_2_O. Similar yellow crystals can be produced (yield = 46%, based on NaL_OEt_). Main IR data (cm^−1^): 2978(m), 1623(s), 1445(m), 1414(m), 1141(s), 1038(s), 933(m), 832(m), 770(m), 722(m), and 582(m). Anal. Calcd for C_42_H_82_Co_2_Ho_2_O_26_P_6_ (%): C, 30.82; H, 5.05. Found: C, 31.01; H, 5.25. UV-Vis (λ_max_/nm with log (ε/dm^3^ mol^−1^ cm^−1^)): 244(4.77) and 335(3.90).

### Preparation of Gd_2_ (L_OEt_)_2_(OAc)_4_ (4)

The preparation of Gd_2_ (L_OEt_)_2_(OAc)_4_ (**4**) followed the same procedure as (**1**), using 27.4 mg (0.062 mmol) Gd(OAc)_3_·6H_2_O. Similar yellow crystals can be produced (yield = 48%, based on NaL_OEt_). Main IR data (cm^−1^): 2977(m), 1624(s), 1446(m), 1415(m), 1142(s), 1039(s), 932(m), 831(m), 771(m), 722(m), and 582(m). Anal. Calcd for C_42_H_82_Co_2_Gd_2_O_26_P_6_ (%): C, 31.11; H, 5.10. Found: C, 31.30; H, 5.28. UV-Vis (λ_max_/nm with log (ε/dm^3^ mol^−1^ cm^−1^)): 243(4.77) and 334(3.89).

## Result and discussion

As mentioned earlier, the organometallic tripodal oxygen ligand NaL_OEt_ is an ideal building block to encapsulate metal cores with its oxygen-based tridentate coordination sites (Gao et al., 2014; Lim et al., 2016; Van Raden et al., 2022). As a result, target lanthanide complexes were synthesized by a one-step reaction of various lanthanide acetate hydrates with NaL_OEt_. Satisfactory crystals for X-ray crystallography can be obtained by evaporating the mixed solution. These air-stable complexes are readily soluble in acetonitrile, acetone, dichloromethane, and methanol. Detailed characterization has been performed by IR spectra, elemental analysis, UV-Vis absorption spectra (Supplementary Figure S1), and magnetic measurements.

### Purity analysis

**FIGURE 1:**
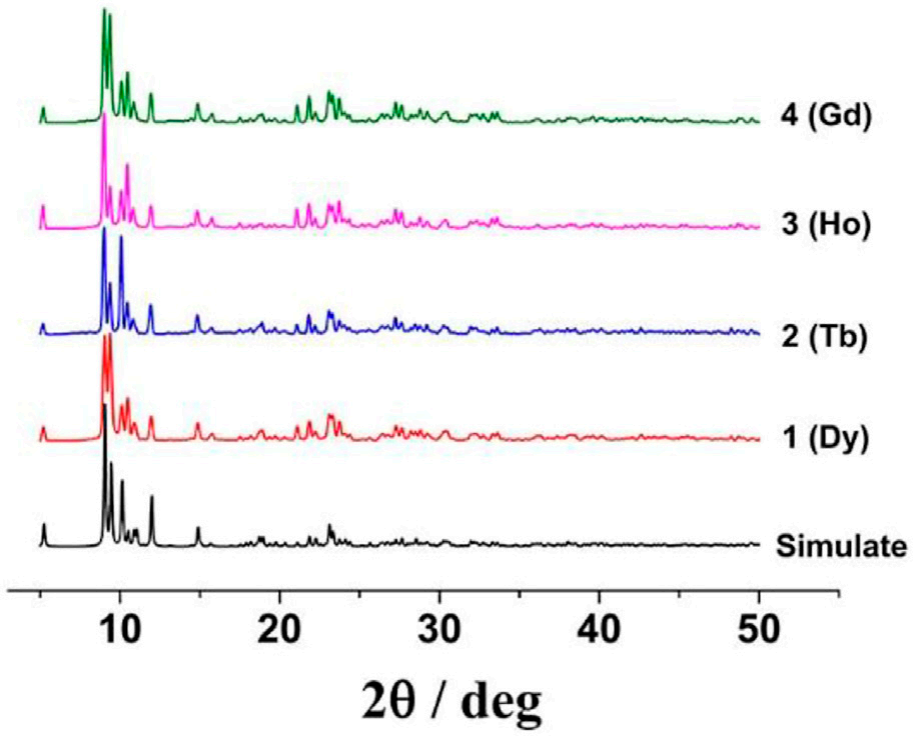
Experimental and simulated PXRD patterns of all complexes.

Powder X-ray diffraction (PXRD) experiment data were measured at room temperature to verify the phase purity of crystal samples. The main experimental peaks match well with the simulated PXRD patterns according to the X-ray crystal data on the respective complexes (Figure 1), confirming the good purity of the prepared products.

### Crystal structure description

**FIGURE 2:**
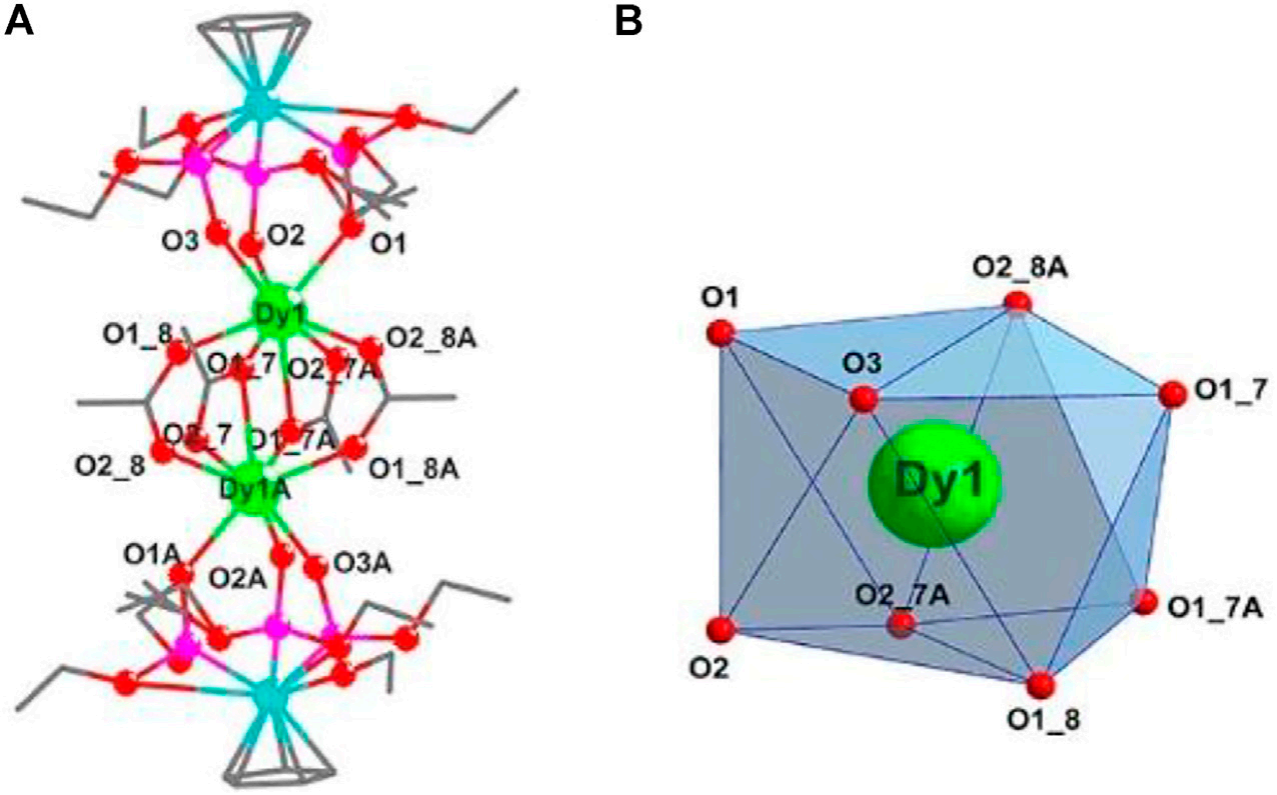
**(A)** Molecular structure diagram of **1** (Dy, green; Co, aqua; O, red; P, pink; and C, gray). The H atoms are omitted for clarity. **(B)** Coordination polyhedron of the Dy1^3+^ ion.

**FIGURE 3:**
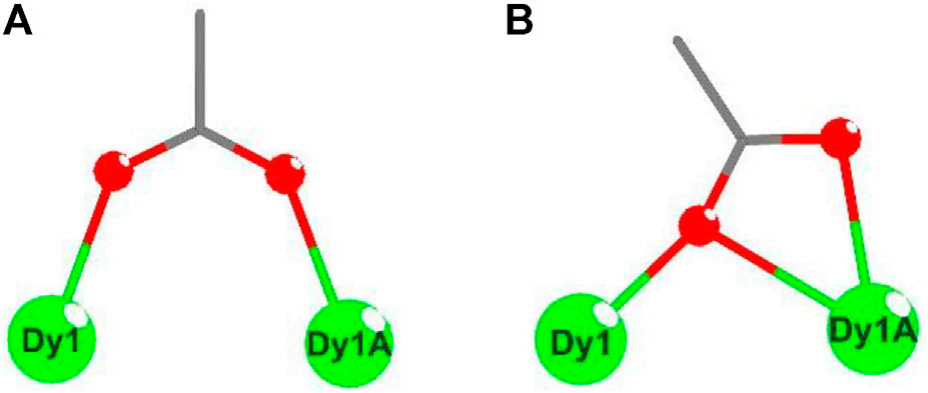
Two coordination patterns μ_2_:η^1^:η^1^ **(A)** and μ_2_:η^1^:η^2^ **(B)** of acetate groups in **1**.

**FIGURE 4:**
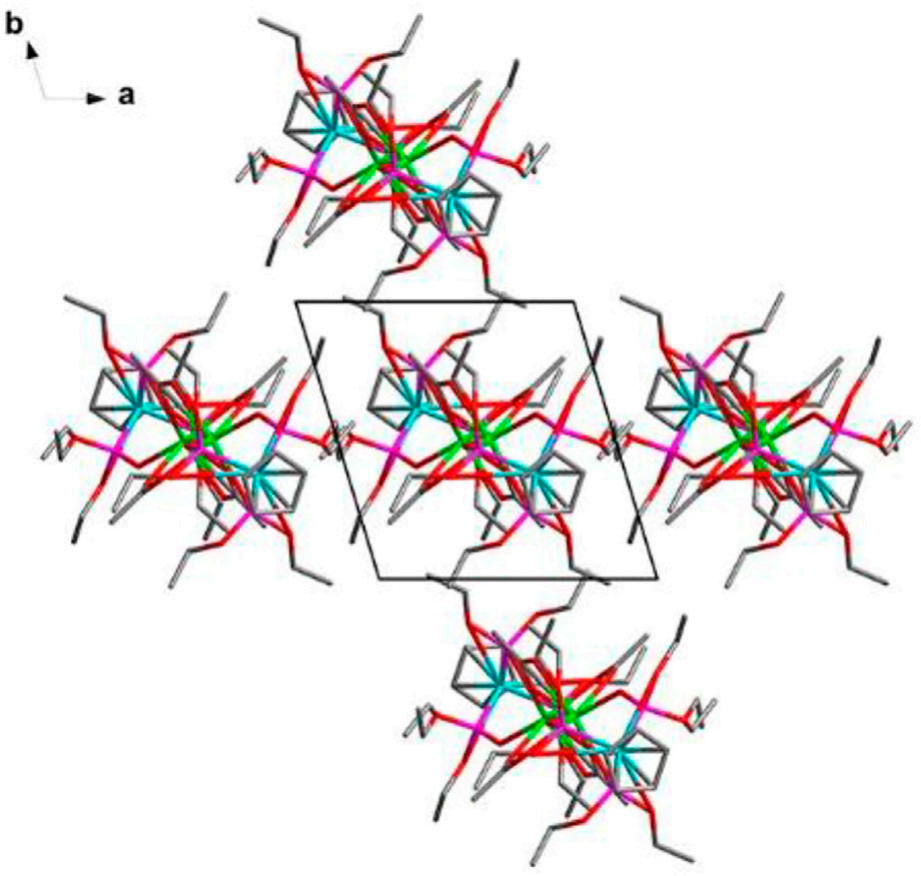
Molecular structure packing diagram along the *c-*axis of **1**.

These electrically neutral complexes crystallize in the triclinic crystal system (*P*ī space group), and each asymmetric unit contains one Ln^3+^ ion, one tripodal anionic ligand [L_OEt_]^−^, and two acetate anions (Figure 2 and Supplementary Figures S2–S4). Related crystallographic parameters are listed in Supplementary Table S1 with CCDC numbers 2182586 (1), 2182587 (2), 2182588 (3), and 2182589 (4). Some main bond length and bond angle data are presented in Supplementary Table S2. Because they are crystallographically isostructural, only the molecular structure of **1** is fully described as a representative. In Figure 2A, the paramagnetic Dy1^3+^ ion is coordinated through eight oxygen atoms (O1, O2, and O3 from tripodal anionic [L_OEt_]^−^, O1_7, O1_8, O1_7A, O2_7A, and O2_8A from acetate anions, respectively). These Dy-O bond lengths range between 2.311 (2) and 2.537 (2) Å. The tripodal anionic [L_OEt_]^−^ situates above the dysprosium (III) ion, and the diamagnetic cobalt (III) ion is surrounded by three phosphorus atoms and a cyclopentadienyl ring. The acetate anions use two different coordination patterns, μ_2_:η^1^:η^1^ (Figure 3A) and μ_2_:η^1^:η^2^ (Figure 3B), to bridge two symmetry-related dysprosium (III) ions with an intramolecular Dy1^3+^···Dy1A^3+^ distance of 3.915 (8) Å. The continuous shape measurement (CShM) method by *SHAPE* analysis (Alvarez et al., 2005) was performed to determine the precise geometry of lanthanide centers (Supplementary Table S3). The eight-coordinated paramagnetic dysprosium (III) ion in **1** has a twisted square antiprism (SAPR, *D*_4d_) conformation (Figure 2B) with calculated CShM value *S* = 1.619. As shown in the crystal packing diagram of **1** (Figure 4), no special intermolecular interactions can be found with the shortest intermolecular distance between dysprosium (III) ions of 9.844 (3) Å.

### Magnetism investigation

**FIGURE 5:**
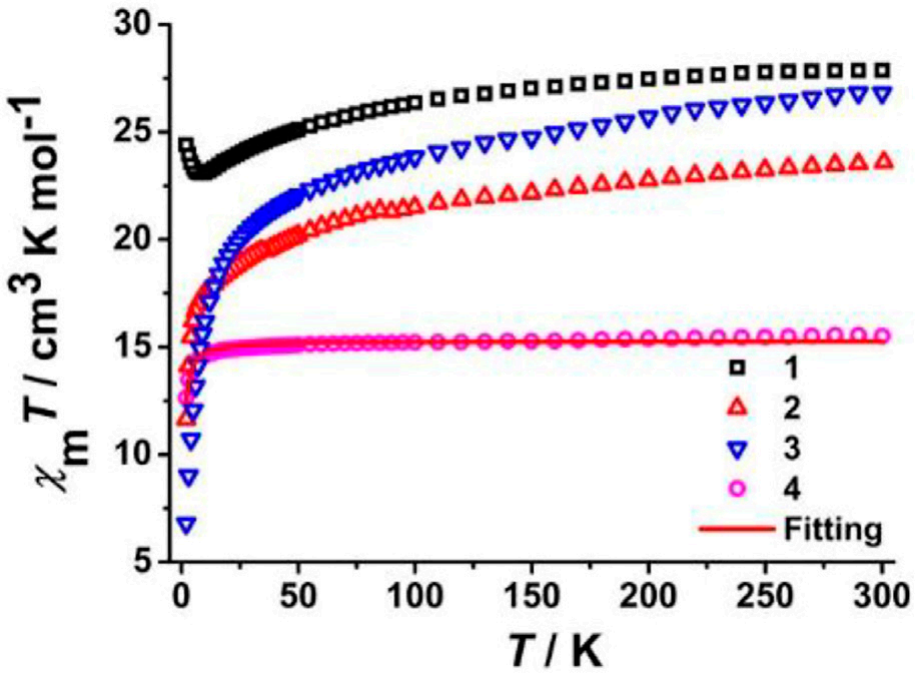
Temperature-dependent *χ*_*M*_ *T* plots under *H*_*dc*_ = 1 kOe of all prepared complexes. The red curve is the fitting result of the experimental *χ*_*M*_ *T* products of **4**.

Temperature-dependent direct current (dc) magnetic susceptibility plots (χ_M_ *T* vs. *T*) are presented in Figure 5, which were collected under *H*_*dc*_ = 1 kOe between 2.0 and 300 K. Since the diamagnetic cobalt (III) cations in the system have no effect on magnetic properties, the measured *χ*_M_ *T* values at 300 K are 27.87 (**1**), 23.60 (**2**), 26.82 (**3**), and 15.51 (**4**) cm^3^ K mol^−1^, comparable to the theoretically calculated results for two isolated paramagnetic lanthanide (III) ions. In high-temperature regions, the *χ*_M_ *T* products of **1**, **2**, and **3** decrease slowly and reach the respective minimum values of 23.08 (**1**, 9.0 K), 11.65 (**2**, 2.0 K), and 6.81 (**3**, 2.0 K) cm^3^ K mol^−1^, usually caused by antiferromagnetic couplings between adjacent lanthanide (III) ions, and/or depopulation of Ln^3+^ ions excited Stark (M_*J*_) sublevels (Lim et al., 2016; Liu et al., 2016; Li J et al., 2021). Upon lowering the temperature to 2.0 K, the *χ*_M_ *T* value of **1** rises again to a maximum of 24.40 cm^3^ K mol^−1^, indicating the existence of weak intramolecular ferromagnetic interactions between the paramagnetic dysprosium (III) centers (Gao et al., 2019; Shen et al., 2020; Li X et al., 2021; Liu et al., 2021). In **4**, the *χ*_*M*_ *T* values are almost unchanged from 300 to 30.0 K and drop eventually to around 2.0 K to 12.64 cm^3^ K mol^−1^, suggesting the occurrence of antiferromagnetic Gd^3+^∙∙∙Gd^3+^ coupling. Subsequently, the isotropic spin Hamiltonian equation *Ĥ* = −2*JŜ*_*Gd1*_ *·Ŝ*_*Gd1A*_ was applied to fit χ_M_ *T* vs. *T* data on **2** in order to reveal the nature and strength of the magnetic interaction between gadolinium (III) ions. The calculated values using the *PHI* program (Chilton et al., 2013a) are *g* = 1.98 and *J* = −0.025 cm^−1^ (the negative *J* value reveals weak antiferromagnetic interactions between gadolinium (III) ions).

**FIGURE 6:**
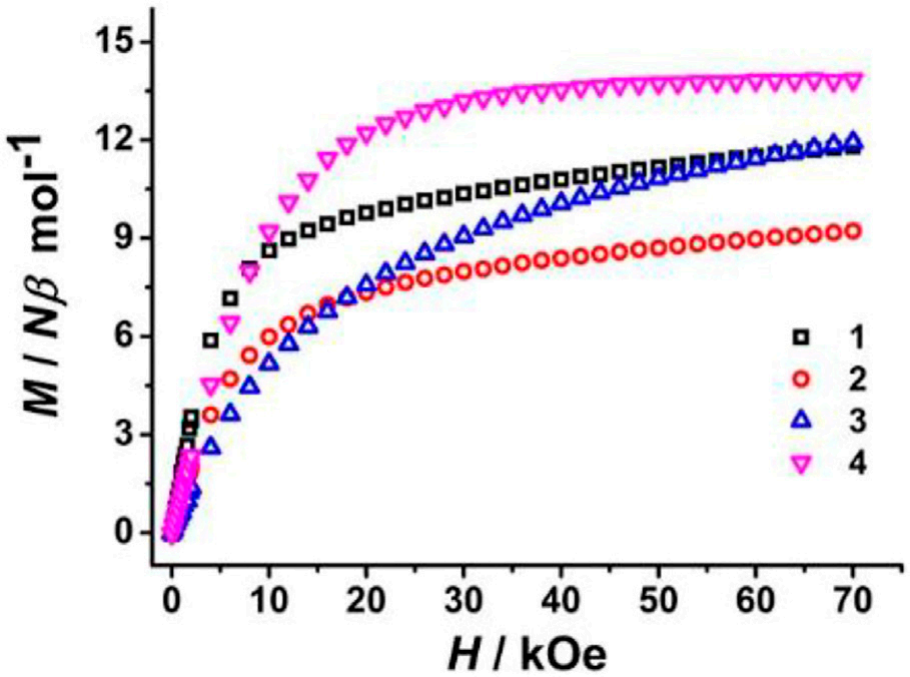
Magnetic field-dependent magnetization plots of all prepared complexes.

Magnetic field-dependent magnetizations (*M* vs. *H*) of all prepared complexes were then measured at 2.0 K under magnetic fields between 0 and 70 kOe, showing that *M* rises rapidly below about 15 kOe and then increases slowly in the high-magnetic field region (Figure 6). The *M* values at *H*_*dc*_ = 70 kOe are 11.80 N*β* for **1**, 9.22 N*β* for **2**, and 11.94 N*β* for **3**. Such deviation from their respective theoretical saturation *M* values is ascribed to crystal field-induced low-excited states and significant magnetic anisotropy (Hutchings et al., 2014; Yang et al., 2014; Gao et al., 2019), while the maximum *M* value of **4** (13.84 N*β*) is consistent with the saturation value of 14.0 N*β* for two noninteracting gadolinium (III) ions.

**FIGURE 7:**
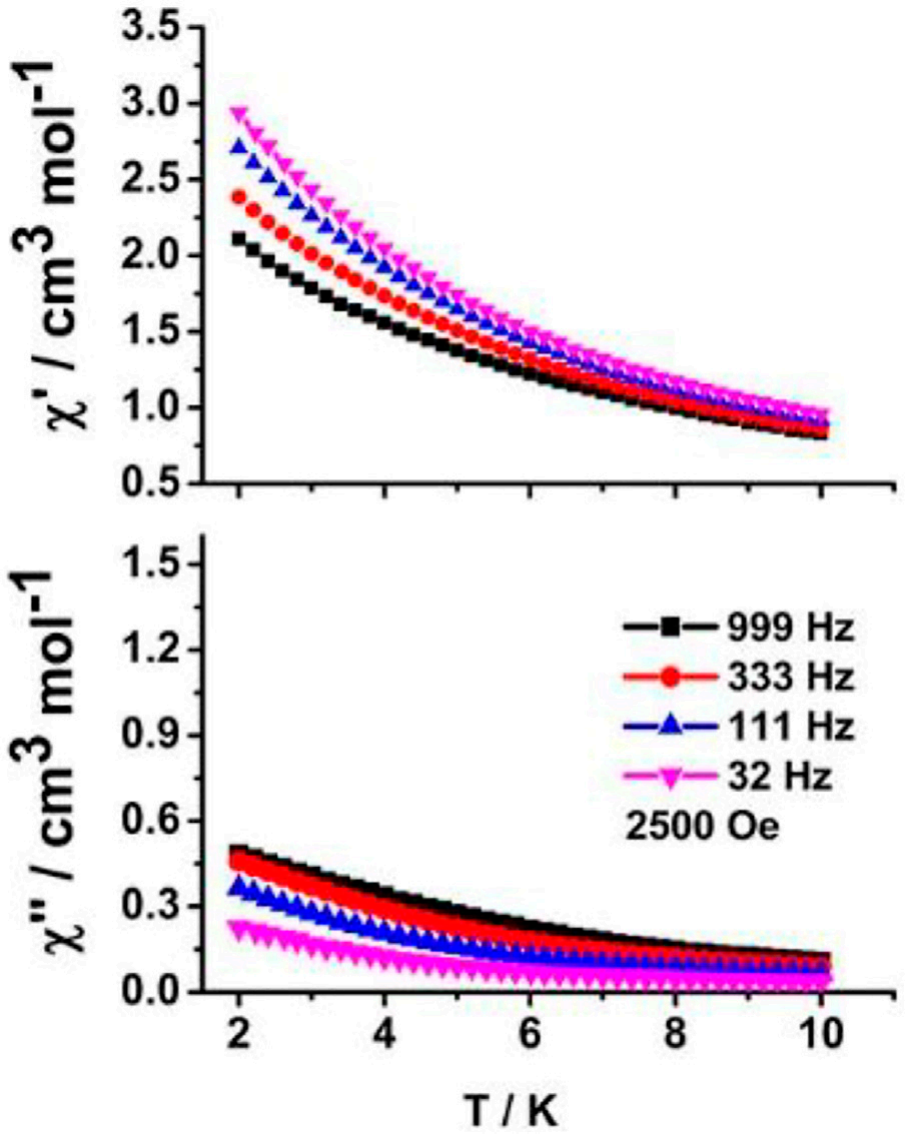
Temperature-dependent in-phase (χ′) (up) and out-of-phase (χ'') (down) ac susceptibility curves under *H*_*dc*_ = 2,500 Oe of **1**.

**FIGURE 8:**
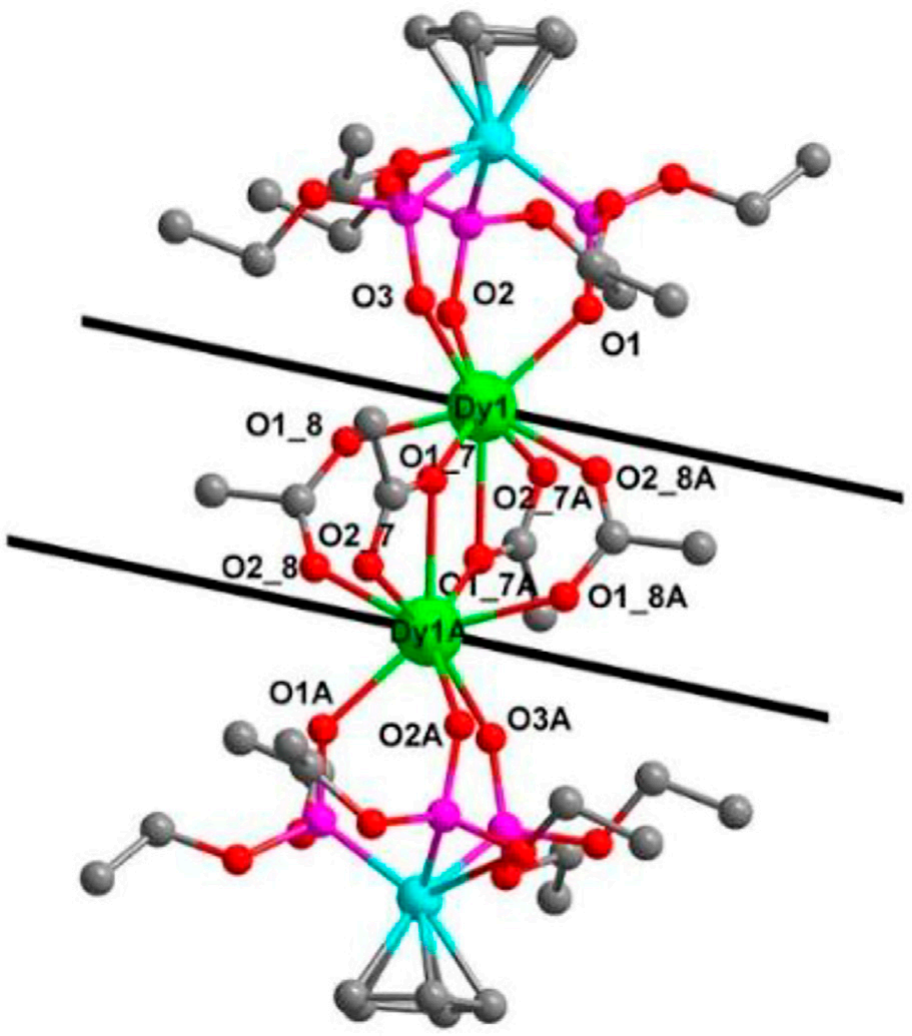
Ground state (M_*J*_ = ±15/2) magnetic anisotropy axes’ direction of dysprosium (III) ion in **1**.

Alternative current (ac) magnetic experiments were measured to study its dynamic magnetic behavior. Unfortunately, no obvious temperature-dependent out-of-phase (*χ''*) susceptibility signal peaks at the high frequency of 999 Hz were shown under *H*_*dc*_ = 0 Oe for **1** containing anisotropic Kramer dysprosium (III) ions (Supplementary Figure S5), possibly originating from the effect of stronger QTM. As a further investigation, the dc magnetic field of 2,500 Oe was employed. The expected *χ''* signal peaks were still unable to be observed (Figure 7). We think there are two main reasons for the lack of SMM behavior in **1**. On the one hand, the relatively larger distortion from the ideal *D*_4d_ geometry around the dysprosium (III) center (higher calculated CShM value *S*_SAPR_ = 1.619) may lead to the weaker ligand-field effect and uniaxial magnetic anisotropy. On the other hand, electrostatic calculation by means of the *MAGELLAN* program (Chilton et al., 2013b) was used to judge the direction of dysprosium (III) ion’s ground state (M_*J*_ = ±15/2) magnetic anisotropy axis (Figure 8). The result shows that the two magnetic axes are parallel to each other in a centrosymmetric molecule, and the angle between the magnetic axis of the Dy1^3+^ ion and the unit vector linking two dysprosium (III) ions (Dy1^3+^ and Dy1A^3+^) is 86.6°. Furthermore, as an important structural parameter affecting the ligand field strength, the Dy1-O2 bond length is 2.311 (2) Å, which is the shortest among those other Dy-O bond lengths in the twisted SAPR polyhedron. The magnetic axis of the Dy1^3+^ ion is aligned along the shortest Dy1-O2 bond with an included angle of 58.4°. The above-mentioned two large angular deviations confirm that such a weak ligand field in this system is not conductive to activating the magnetic relaxation process.

For complexes **2** and **3** containing anisotropic non-Kramer lanthanide (III) ions, the χ'' signals were also very weak at the frequency of 999 Hz under *H*_*dc*_ = 0 Oe and *H*_*dc*_ = 2,500 Oe, respectively (Supplementary Figures S6, S7), which is raised by their fast magnetization relaxation behavior in such a weak ligand field environment.

## Conclusion

In this work, we reported four acetate-bridged homodinuclear lanthanide complexes based on a tripodal oxygen ligand NaL_OEt_. Structural analyses show acetate anions bridge two symmetry-related Ln^3+^ ions in the μ_2_:η^1^:η^1^ and μ_2_:η^1^:η^2^ coordination patterns, and each lanthanide ion owns a twisted SAPR conformation. Magnetic analyses reveal the weak intramolecular ferromagnetic interaction between dysprosium (III) ions in **1** and antiferromagnetic Ln^3+^···Ln^3+^ coupling in the other complexes. The weaker ligand-field effect caused by the larger distorted geometry and the deviation of the magnetic anisotropy axis orientation with a specific lanthanide–ligand coordination bond leads to the lack of SMM behavior.

Although the expected SMM behaviors could not be found in this system, these well-known factors, including the effective suppression of QTM, regulation of Ln^3+^···Ln^3+^ magnetic interactions, and construction of reasonable crystal field symmetry, still remain important for affecting the slow magnetic relaxation behaviors of SMMs. Further efforts to design, synthesize, and study novel molecular magnetic materials are in progress in our group.

## Data Availability

The datasets presented in this study can be found in online repositories. The names of the repository/repositories and accession number(s) can be found in the article/Supplementary Material.
