# Supplementary material for: Syntheses, structures, and magnetic properties of acetate-bridged lanthanide complexes based on a tripodal oxygen ligand
Source: Front Chem. 2022 Sep 19;10:1021358. doi: 10.3389/fchem.2022.1021358 (PMC9527289; doi:10.3389/fchem.2022.1021358)

checkCIF/PLATON report

Structure factors have been supplied for datablock(s) GF20180430B_0m, GF20180607A_0m_a, GF20180607C_0m, GF20180607D_0m

THIS REPORT IS FOR GUIDANCE ONLY. IF USED AS PART OF A REVIEW PROCEDURE FOR PUBLICATION, IT SHOULD NOT REPLACE THE EXPERTISE OF AN EXPERIENCED CRYSTALLOGRAPHIC REFEREE.

No syntax errors found. CIF dictionary Interpreting this report

Datablock: GF20180430B_0m

```
Bond precision:   C-C = 0.0071 A          Wavelength=0.71073

Cell:            a=9.8444(10)       b=10.1916(10)     c=17.0109(17)
                 alpha=92.749(1)    beta=97.058(1)    gamma=106.308(1)
Temperature:     296 K

                  Calculated                   Reported
Volume            1619.5(3)                    1619.5(3)
Space group       P -1                         P -1
Hall group        -P 1                         -P 1
Moiety formula    C42 H82 Co2 Dy2 O26 P6       ?
Sum formula       C42 H82 Co2 Dy2 O26 P6       C42 H82 Co2 Dy2 O26 P6
Mr                1631.76                      1631.75
Dx,g cm-3         1.673                        1.673
Z                 1                            1
Mu (mm-1)         3.005                        3.005
F000              818.0                        818.0
F000'             819.29
h,k,lmax          12,12,20                     12,12,20
Nref              6375                         6216
Tmin,Tmax         0.554,0.548                  0.545,0.553
Tmin'             0.543

Correction method= # Reported T Limits: Tmin=0.545 Tmax=0.553
AbsCorr = MULTI-SCAN

Data completeness= 0.975           Theta(max)= 26.022

R(reflections)= 0.0271( 5689)                      wR2(reflections)= 0.0713( 6216)

S = 1.044                   Npar= 391
```

The following ALERTS were generated. Each ALERT has the format
test-name_ALERT_alert-type_alert-level.
Click on the hyperlinks for more details of the test.

Alert level C

```
PLAT213_ALERT_2_C Atom C2_1            has ADP max/min Ratio .....         3.2 prolat
PLAT220_ALERT_2_C NonSolvent   Resd 1  C   Ueq(max)/Ueq(min) Range         4.2 Ratio
PLAT242_ALERT_2_C Low    'MainMol' Ueq as Compared to Neighbors of         Co1 Check
PLAT242_ALERT_2_C Low    'MainMol' Ueq as Compared to Neighbors of        C1_1 Check
PLAT242_ALERT_2_C Low    'MainMol' Ueq as Compared to Neighbors of        C1_7 Check
PLAT242_ALERT_2_C Low    'MainMol' Ueq as Compared to Neighbors of        C1_8 Check
PLAT360_ALERT_2_C Short  C(sp3)-C(sp3) Bond  C1_5     - C2_5     .        1.40 Ang.
PLAT910_ALERT_3_C Missing # of FCF Reflection(s) Below Theta(Min).           6 Note
PLAT911_ALERT_3_C Missing FCF Refl Between Thmin & STh/L=    0.600         108 Report
PLAT913_ALERT_3_C Missing # of Very Strong Reflections in FCF ....           4 Note
```

Alert level G

```
PLAT002_ALERT_2_G Number of Distance or Angle Restraints on AtSite          10 Note
PLAT003_ALERT_2_G Number of Uiso or Uij Restrained non-H Atoms ...          10 Report
PLAT066_ALERT_1_G Predicted and Reported Tmin&Tmax Range Identical           ? Check
PLAT154_ALERT_1_G The s.u.'s on the Cell Angles are Equal ..(Note)       0.001 Degree
PLAT175_ALERT_4_G The CIF-Embedded .res File Contains SAME Records           3 Report
PLAT177_ALERT_4_G The CIF-Embedded .res File Contains DELU Records           2 Report
PLAT178_ALERT_4_G The CIF-Embedded .res File Contains SIMU Records           2 Report
PLAT232_ALERT_2_G Hirshfeld Test Diff (M-X)  Dy1      --O1_7_a   .         6.1 s.u.
PLAT232_ALERT_2_G Hirshfeld Test Diff (M-X)  Dy1      --O2_7_a   .         5.3 s.u.
PLAT301_ALERT_3_G Main Residue  Disorder .............(Resd  1  )          10% Note
PLAT720_ALERT_4_G Number of Unusual/Non-Standard Labels ..........          76 Note
PLAT793_ALERT_4_G Model has Chirality at P1          (Centro SPGR)           S Verify
PLAT860_ALERT_3_G Number of Least-Squares Restraints .............         126 Note
PLAT883_ALERT_1_G No Info/Value for _atom_sites_solution_primary .     Please Do !
PLAT912_ALERT_4_G Missing # of FCF Reflections Above STh/L=  0.600          45 Note
PLAT933_ALERT_2_G Number of HKL-OMIT Records in Embedded .res File           5 Note
PLAT941_ALERT_3_G Average HKL Measurement Multiplicity ...........         1.4 Low
PLAT965_ALERT_2_G The SHELXL WEIGHT Optimisation has not Converged     Please Check
PLAT978_ALERT_2_G Number C-C Bonds with Positive Residual Density.           1 Info
```

0 **ALERT level A** = Most likely a serious problem - resolve or explain
0 **ALERT level B** = A potentially serious problem, consider carefully
10 **ALERT level C** = Check. Ensure it is not caused by an omission or oversight
19 **ALERT level G** = General information/check it is not something unexpected

3 ALERT type 1 CIF construction/syntax error, inconsistent or missing data
14 ALERT type 2 Indicator that the structure model may be wrong or deficient
6 ALERT type 3 Indicator that the structure quality may be low
6 ALERT type 4 Improvement, methodology, query or suggestion
0 ALERT type 5 Informative message, check

Datablock: GF20180607A_0m_a

Bond precision: C-C = 0.0067 A Wavelength=0.71073

Cell:	a=9.8434(6)	b=10.1878(7)	c=17.0374(11)
	alpha=92.807(1)	beta=96.990(1)	gamma=106.268(1)
Temperature:	296 K		

	Calculated	Reported
Volume	1621.76(18)	1621.76(18)
Space group	P -1	P -1
Hall group	-P 1	-P 1
Moiety formula	C42 H82 Co2 O26 P6 Tb2	?
Sum formula	C42 H82 Co2 O26 P6 Tb2	C42 H82 Co2 O26 P6 Tb2
Mr	1624.62	1624.59
Dx,g cm-3	1.663	1.663
Z	1	1
Mu (mm-1)	2.877	2.877
F000	816.0	816.0
F000'	817.32	
h,k,lmax	12,12,21	12,12,21
Nref	6382	6238
Tmin,Tmax	0.568,0.562	0.558,0.569
Tmin'	0.557	

Correction method= # Reported T Limits: Tmin=0.558 Tmax=0.569
AbsCorr = MULTI-SCAN

Data completeness= 0.977 Theta(max)= 26.021

R(reflections)= 0.0272(5707) wR2(reflections)= 0.0725(6238)

S = 1.044 Npar= 391

The following ALERTS were generated. Each ALERT has the format
test-name_ALERT_alert-type_alert-level.
Click on the hyperlinks for more details of the test.

Alert level C

```
PLAT213_ALERT_2_C Atom C2_1             has ADP max/min Ratio .....        3.3 prolat
PLAT220_ALERT_2_C NonSolvent   Resd 1  C   Ueq(max)/Ueq(min) Range        4.3 Ratio
PLAT222_ALERT_3_C NonSolvent Resd 1   H   Uiso(max)/Uiso(min) Range       4.2 Ratio
PLAT242_ALERT_2_C Low    'MainMol' Ueq as Compared to Neighbors of        Co1 Check
PLAT242_ALERT_2_C Low    'MainMol' Ueq as Compared to Neighbors of       C1_1 Check
PLAT242_ALERT_2_C Low    'MainMol' Ueq as Compared to Neighbors of       C1_7 Check
PLAT242_ALERT_2_C Low    'MainMol' Ueq as Compared to Neighbors of       C1_8 Check
PLAT360_ALERT_2_C Short  C(sp3)-C(sp3) Bond  C1_1     - C2_1     .       1.42 Ang.
PLAT360_ALERT_2_C Short  C(sp3)-C(sp3) Bond  C1_5     - C2_5     .       1.39 Ang.
```

```
PLAT910_ALERT_3_C Missing # of FCF Reflection(s) Below Theta(Min).          7 Note
PLAT911_ALERT_3_C Missing FCF Refl Between Thmin & STh/L=    0.600        101 Report
PLAT913_ALERT_3_C Missing # of Very Strong Reflections in FCF ....          4 Note
```

Alert level G

```
PLAT002_ALERT_2_G Number of Distance or Angle Restraints on AtSite         10 Note
PLAT003_ALERT_2_G Number of Uiso or Uij Restrained non-H Atoms ...         10 Report
PLAT066_ALERT_1_G Predicted and Reported Tmin&Tmax Range Identical          ? Check
PLAT154_ALERT_1_G The s.u.'s on the Cell Angles are Equal ..(Note)      0.001 Degree
PLAT175_ALERT_4_G The CIF-Embedded .res File Contains SAME Records          3 Report
PLAT177_ALERT_4_G The CIF-Embedded .res File Contains DELU Records          2 Report
PLAT178_ALERT_4_G The CIF-Embedded .res File Contains SIMU Records          2 Report
PLAT232_ALERT_2_G Hirshfeld Test Diff (M-X)  Tb1      --O1_7_a   .        7.1 s.u.
PLAT232_ALERT_2_G Hirshfeld Test Diff (M-X)  Tb1      --O2_7_a   .        5.9 s.u.
PLAT301_ALERT_3_G Main Residue  Disorder .............(Resd  1  )         10% Note
PLAT720_ALERT_4_G Number of Unusual/Non-Standard Labels ..........         76 Note
PLAT793_ALERT_4_G Model has Chirality at P1         (Centro SPGR)          S Verify
PLAT860_ALERT_3_G Number of Least-Squares Restraints ............         126 Note
PLAT883_ALERT_1_G No Info/Value for _atom_sites_solution_primary .     Please Do !
PLAT912_ALERT_4_G Missing # of FCF Reflections Above STh/L=  0.600         37 Note
PLAT933_ALERT_2_G Number of HKL-OMIT Records in Embedded .res File          3 Note
PLAT941_ALERT_3_G Average HKL Measurement Multiplicity ..........         1.4 Low
PLAT978_ALERT_2_G Number C-C Bonds with Positive Residual Density.          2 Info
```

---

0 **ALERT level A** = Most likely a serious problem - resolve or explain
0 **ALERT level B** = A potentially serious problem, consider carefully
12 **ALERT level C** = Check. Ensure it is not caused by an omission or oversight
18 **ALERT level G** = General information/check it is not something unexpected

3 ALERT type 1 CIF construction/syntax error, inconsistent or missing data
14 ALERT type 2 Indicator that the structure model may be wrong or deficient
7 ALERT type 3 Indicator that the structure quality may be low
6 ALERT type 4 Improvement, methodology, query or suggestion
0 ALERT type 5 Informative message, check

Datablock: GF20180607D_0m

```
Bond precision:   C-C = 0.0079 A          Wavelength=0.71073

Cell:            a=9.8488(9)        b=10.1850(9)       c=16.9611(15)
                 alpha=92.735(1)    beta=97.128(1)     gamma=106.407(1)
Temperature:     296 K
```

	Calculated	Reported
Volume	1613.3(3)	1613.3(2)
Space group	P -1	P -1
Hall group	-P 1	-P 1
Moiety formula	C42 H82 Co2 Ho2 O26 P6	?
Sum formula	C42 H82 Co2 Ho2 O26 P6	C42 H82 Co2 Ho2 O26 P6
Mr	1636.62	1636.61
Dx,g cm-3	1.685	1.684
Z	1	1
Mu (mm-1)	3.152	3.152
F000	820.0	820.0
F000'	821.23	
h,k,lmax	12,12,21	12,12,21
Nref	6596	6428
Tmin,Tmax	0.538,0.532	0.529,0.537
Tmin'	0.527	

Correction method= # Reported T Limits: Tmin=0.529 Tmax=0.537
AbsCorr = MULTI-SCAN

Data completeness= 0.975 Theta(max)= 26.371

R(reflections)= 0.0318(5702) wR2(reflections)= 0.0817(6428)

S = 1.009 Npar= 391

The following ALERTS were generated. Each ALERT has the format
test-name_ALERT_alert-type_alert-level.
Click on the hyperlinks for more details of the test.

Alert level C

```
PLAT213_ALERT_2_C Atom C2_1            has ADP max/min Ratio .....        3.5 prolat
PLAT220_ALERT_2_C NonSolvent   Resd 1  C   Ueq(max)/Ueq(min) Range        4.2 Ratio
PLAT242_ALERT_2_C Low   'MainMol' Ueq as Compared to Neighbors of         Co1 Check
PLAT242_ALERT_2_C Low   'MainMol' Ueq as Compared to Neighbors of        C1_1 Check
PLAT242_ALERT_2_C Low   'MainMol' Ueq as Compared to Neighbors of        C1_7 Check
PLAT242_ALERT_2_C Low   'MainMol' Ueq as Compared to Neighbors of        C1_8 Check
PLAT360_ALERT_2_C Short  C(sp3)-C(sp3) Bond  C1_1     - C2_1     .       1.43 Ang.
PLAT360_ALERT_2_C Short  C(sp3)-C(sp3) Bond  C1_5     - C2_5     .       1.39 Ang.
PLAT910_ALERT_3_C Missing # of FCF Reflection(s) Below Theta(Min).          8 Note
PLAT911_ALERT_3_C Missing FCF Refl Between Thmin & STh/L=    0.600         96 Report
PLAT913_ALERT_3_C Missing # of Very Strong Reflections in FCF ....          4 Note
PLAT977_ALERT_2_C Check Negative Difference Density on H1A_5     .      -0.41 eA-3
```

Alert level G

```
PLAT002_ALERT_2_G Number of Distance or Angle Restraints on AtSite         10 Note
PLAT003_ALERT_2_G Number of Uiso or Uij Restrained non-H Atoms ...         10 Report
```

```
PLAT066_ALERT_1_G Predicted and Reported Tmin&Tmax Range Identical          ? Check
PLAT154_ALERT_1_G The s.u.'s on the Cell Angles are Equal ..(Note)      0.001 Degree
PLAT175_ALERT_4_G The CIF-Embedded .res File Contains SAME Records          3 Report
PLAT177_ALERT_4_G The CIF-Embedded .res File Contains DELU Records          2 Report
PLAT178_ALERT_4_G The CIF-Embedded .res File Contains SIMU Records          2 Report
PLAT232_ALERT_2_G Hirshfeld Test Diff (M-X)  Ho1      --O1_7_a   .        6.3 s.u.
PLAT232_ALERT_2_G Hirshfeld Test Diff (M-X)  Ho1      --O2_7_a   .        5.5 s.u.
PLAT301_ALERT_3_G Main Residue  Disorder .............(Resd  1  )         10% Note
PLAT720_ALERT_4_G Number of Unusual/Non-Standard Labels ..........         76 Note
PLAT793_ALERT_4_G Model has Chirality at P1         (Centro SPGR)           S Verify
PLAT860_ALERT_3_G Number of Least-Squares Restraints ............         126 Note
PLAT883_ALERT_1_G No Info/Value for _atom_sites_solution_primary .     Please Do !
PLAT912_ALERT_4_G Missing # of FCF Reflections Above STh/L=  0.600         64 Note
PLAT933_ALERT_2_G Number of HKL-OMIT Records in Embedded .res File          2 Note
PLAT941_ALERT_3_G Average HKL Measurement Multiplicity ...........        1.4 Low
PLAT965_ALERT_2_G The SHELXL WEIGHT Optimisation has not Converged     Please Check
PLAT978_ALERT_2_G Number C-C Bonds with Positive Residual Density.          1 Info
```

---

   0 **ALERT level A** = Most likely a serious problem - resolve or explain
   0 **ALERT level B** = A potentially serious problem, consider carefully
  12 **ALERT level C** = Check. Ensure it is not caused by an omission or oversight
  19 **ALERT level G** = General information/check it is not something unexpected

   3 ALERT type 1 CIF construction/syntax error, inconsistent or missing data
  16 ALERT type 2 Indicator that the structure model may be wrong or deficient
   6 ALERT type 3 Indicator that the structure quality may be low
   6 ALERT type 4 Improvement, methodology, query or suggestion
   0 ALERT type 5 Informative message, check

Datablock: GF20180607C_0m

```
Bond precision:   C-C = 0.0074 A           Wavelength=0.71073

Cell:            a=9.8497(7)        b=10.1837(8)       c=17.0879(13)
                 alpha=92.808(1)    beta=96.985(1)     gamma=106.228(1)
Temperature:     296 K
```

	Calculated	Reported
Volume	1627.3(2)	1627.3(2)
Space group	P -1	P -1
Hall group	-P 1	-P 1
Moiety formula	C42 H82 Co2 Gd2 O26 P6	?
Sum formula	C42 H82 Co2 Gd2 O26 P6	C42 H82 Co2 Gd2 O26 P6
Mr	1621.26	1621.25
Dx,g cm-3	1.654	1.654
Z	1	1
Mu (mm-1)	2.732	2.732
F000	814.0	814.0
F000'	815.32	
h,k,lmax	12,12,21	12,12,21
Nref	6650	6478
Tmin,Tmax	0.585,0.579	0.572,0.586
Tmin'	0.573	

Correction method= # Reported T Limits: Tmin=0.572 Tmax=0.586
AbsCorr = MULTI-SCAN

Data completeness= 0.974 Theta(max)= 26.369

R(reflections)= 0.0293(5803) wR2(reflections)= 0.0785(6478)

S = 0.984 Npar= 391

The following ALERTS were generated. Each ALERT has the format
test-name_ALERT_alert-type_alert-level.
Click on the hyperlinks for more details of the test.

Alert level C

```
PLAT213_ALERT_2_C Atom C2_1            has ADP max/min Ratio .....        3.4 prolat
PLAT220_ALERT_2_C NonSolvent   Resd 1  C   Ueq(max)/Ueq(min) Range        4.3 Ratio
PLAT222_ALERT_3_C NonSolvent Resd 1  H   Uiso(max)/Uiso(min) Range        4.1 Ratio
PLAT242_ALERT_2_C Low    'MainMol' Ueq as Compared to Neighbors of        Co1 Check
PLAT242_ALERT_2_C Low    'MainMol' Ueq as Compared to Neighbors of         P1 Check
PLAT242_ALERT_2_C Low    'MainMol' Ueq as Compared to Neighbors of       C1_1 Check
PLAT242_ALERT_2_C Low    'MainMol' Ueq as Compared to Neighbors of       C1_7 Check
PLAT242_ALERT_2_C Low    'MainMol' Ueq as Compared to Neighbors of       C1_8 Check
PLAT360_ALERT_2_C Short  C(sp3)-C(sp3) Bond  C1_1     - C2_1     .       1.43 Ang.
PLAT360_ALERT_2_C Short  C(sp3)-C(sp3) Bond  C1_5     - C2_5     .       1.39 Ang.
PLAT911_ALERT_3_C Missing FCF Refl Between Thmin & STh/L=    0.600         98 Report
PLAT913_ALERT_3_C Missing # of Very Strong Reflections in FCF ....          4 Note
PLAT977_ALERT_2_C Check Negative Difference Density on H1A_5     .      -0.35 eA-3
```

Alert level G

```
PLAT002_ALERT_2_G Number of Distance or Angle Restraints on AtSite         10 Note
```

```
PLAT003_ALERT_2_G Number of Uiso or Uij Restrained non-H Atoms ...         10 Report
PLAT066_ALERT_1_G Predicted and Reported Tmin&Tmax Range Identical          ? Check
PLAT154_ALERT_1_G The s.u.'s on the Cell Angles are Equal ..(Note)      0.001 Degree
PLAT175_ALERT_4_G The CIF-Embedded .res File Contains SAME Records          3 Report
PLAT177_ALERT_4_G The CIF-Embedded .res File Contains DELU Records          2 Report
PLAT178_ALERT_4_G The CIF-Embedded .res File Contains SIMU Records          2 Report
PLAT232_ALERT_2_G Hirshfeld Test Diff (M-X)  Gd1      --O1_7_a   .        6.9 s.u.
PLAT301_ALERT_3_G Main Residue  Disorder .............(Resd  1  )         10% Note
PLAT720_ALERT_4_G Number of Unusual/Non-Standard Labels ..........         76 Note
PLAT793_ALERT_4_G Model has Chirality at P1         (Centro SPGR)           S Verify
PLAT860_ALERT_3_G Number of Least-Squares Restraints ............         126 Note
PLAT883_ALERT_1_G No Info/Value for _atom_sites_solution_primary .     Please Do !
PLAT910_ALERT_3_G Missing # of FCF Reflection(s) Below Theta(Min).          3 Note
PLAT912_ALERT_4_G Missing # of FCF Reflections Above STh/L=  0.600         71 Note
PLAT933_ALERT_2_G Number of HKL-OMIT Records in Embedded .res File          4 Note
PLAT941_ALERT_3_G Average HKL Measurement Multiplicity ..........         1.4 Low
PLAT965_ALERT_2_G The SHELXL WEIGHT Optimisation has not Converged     Please Check
PLAT978_ALERT_2_G Number C-C Bonds with Positive Residual Density.          1 Info
```

---

0 **ALERT level A** = Most likely a serious problem - resolve or explain
0 **ALERT level B** = A potentially serious problem, consider carefully
13 **ALERT level C** = Check. Ensure it is not caused by an omission or oversight
19 **ALERT level G** = General information/check it is not something unexpected

3 ALERT type 1 CIF construction/syntax error, inconsistent or missing data
16 ALERT type 2 Indicator that the structure model may be wrong or deficient
7 ALERT type 3 Indicator that the structure quality may be low
6 ALERT type 4 Improvement, methodology, query or suggestion
0 ALERT type 5 Informative message, check

It is advisable to attempt to resolve as many as possible of the alerts in all categories. Often the minor alerts point to easily fixed oversights, errors and omissions in your CIF or refinement strategy, so attention to these fine details can be worthwhile. In order to resolve some of the more serious problems it may be necessary to carry out additional measurements or structure refinements. However, the purpose of your study may justify the reported deviations and the more serious of these should normally be commented upon in the discussion or experimental section of a paper or in the "special_details" fields of the CIF. checkCIF was carefully designed to identify outliers and unusual parameters, but every test has its limitations and alerts that are not important in a particular case may appear. Conversely, the absence of alerts does not guarantee there are no aspects of the results needing attention. It is up to the individual to critically assess their own results and, if necessary, seek expert advice.

Publication of your CIF in IUCr journals

A basic structural check has been run on your CIF. These basic checks will be run on all CIFs submitted for publication in IUCr journals (*Acta Crystallographica*, *Journal of Applied Crystallography*, *Journal of Synchrotron Radiation*); however, if you intend to submit to *Acta Crystallographica Section C* or *E* or *IUCrData*, you should make sure that full publication checks are run on the final version of your CIF prior to submission.

Publication of your CIF in other journals

Please refer to the *Notes for Authors* of the relevant journal for any special instructions relating to CIF submission.

PLATON version of 18/05/2022; check.def file version of 17/05/2022

Datablock GF20180430B_0m - ellipsoid plot

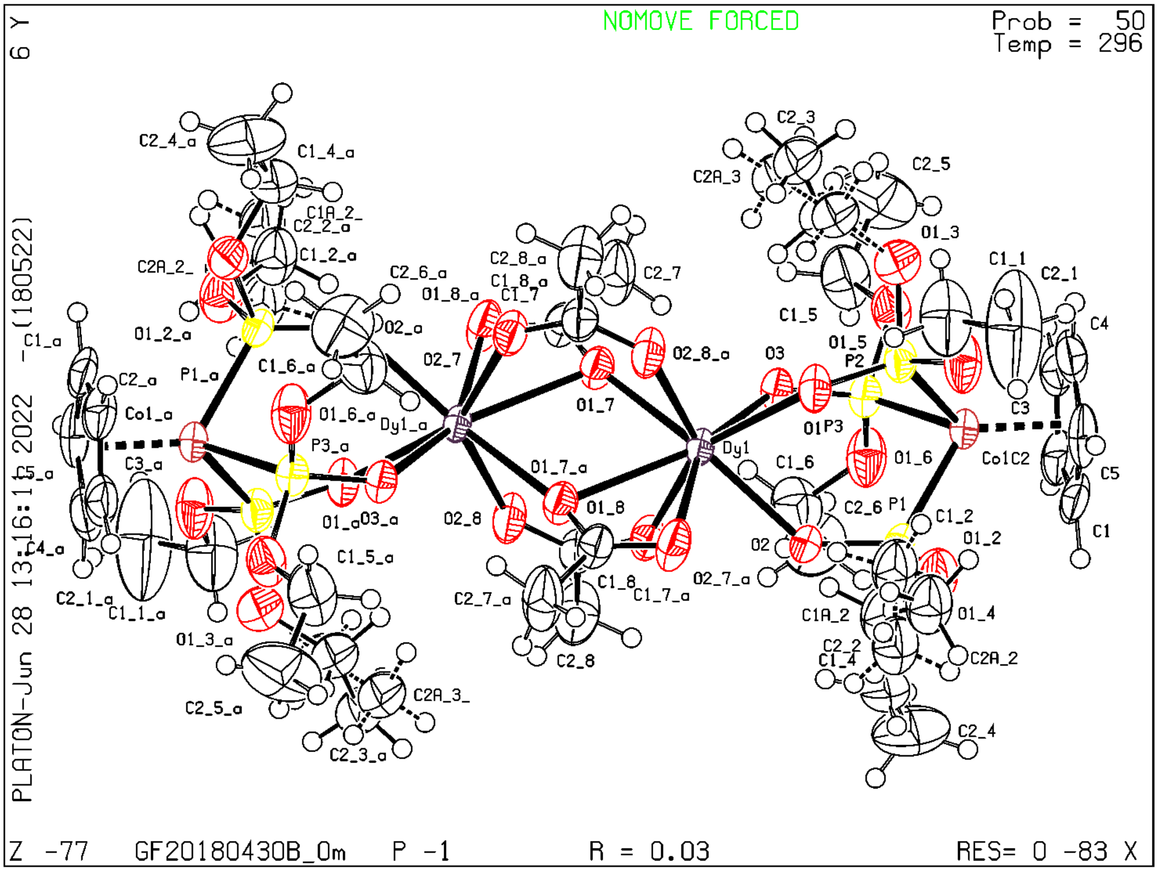

Datablock GF20180607A_0m_a - ellipsoid plot

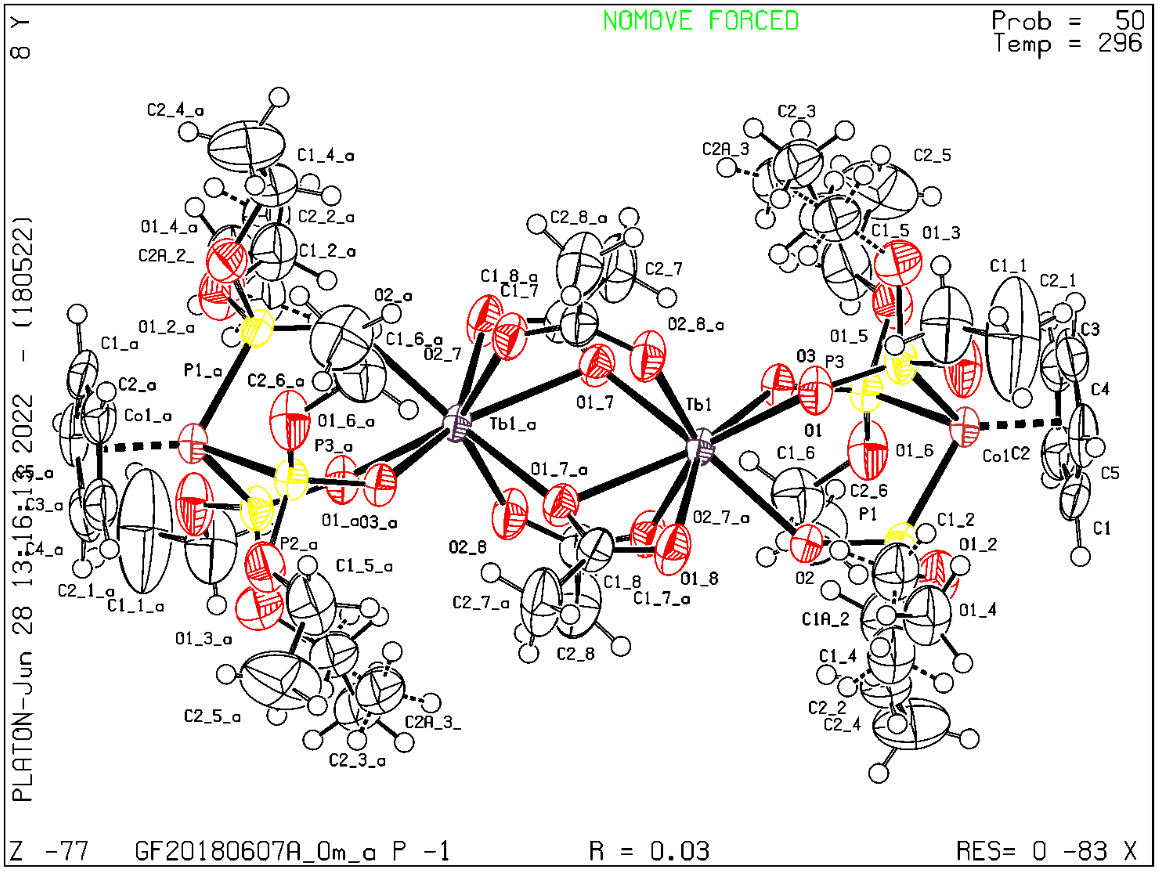

Datablock GF20180607D_0m - ellipsoid plot

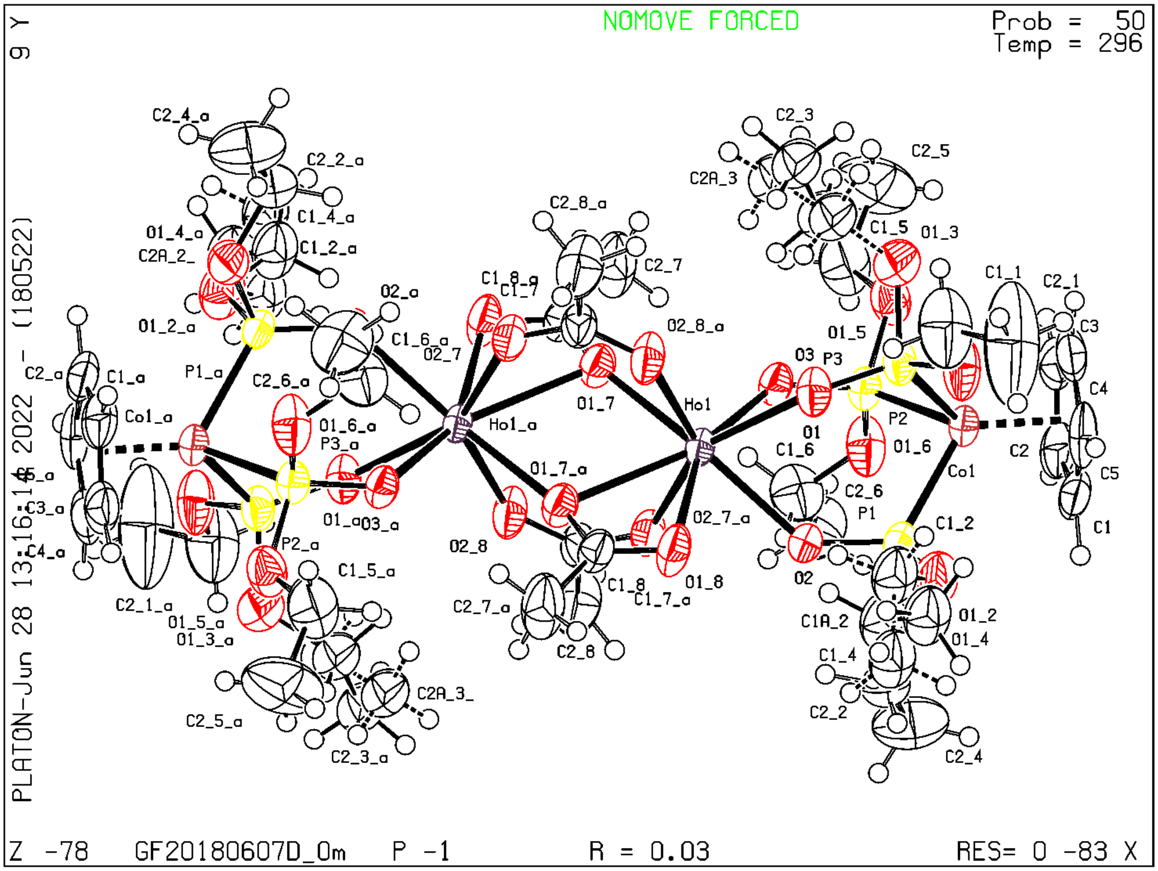

Datablock GF20180607C_0m - ellipsoid plot

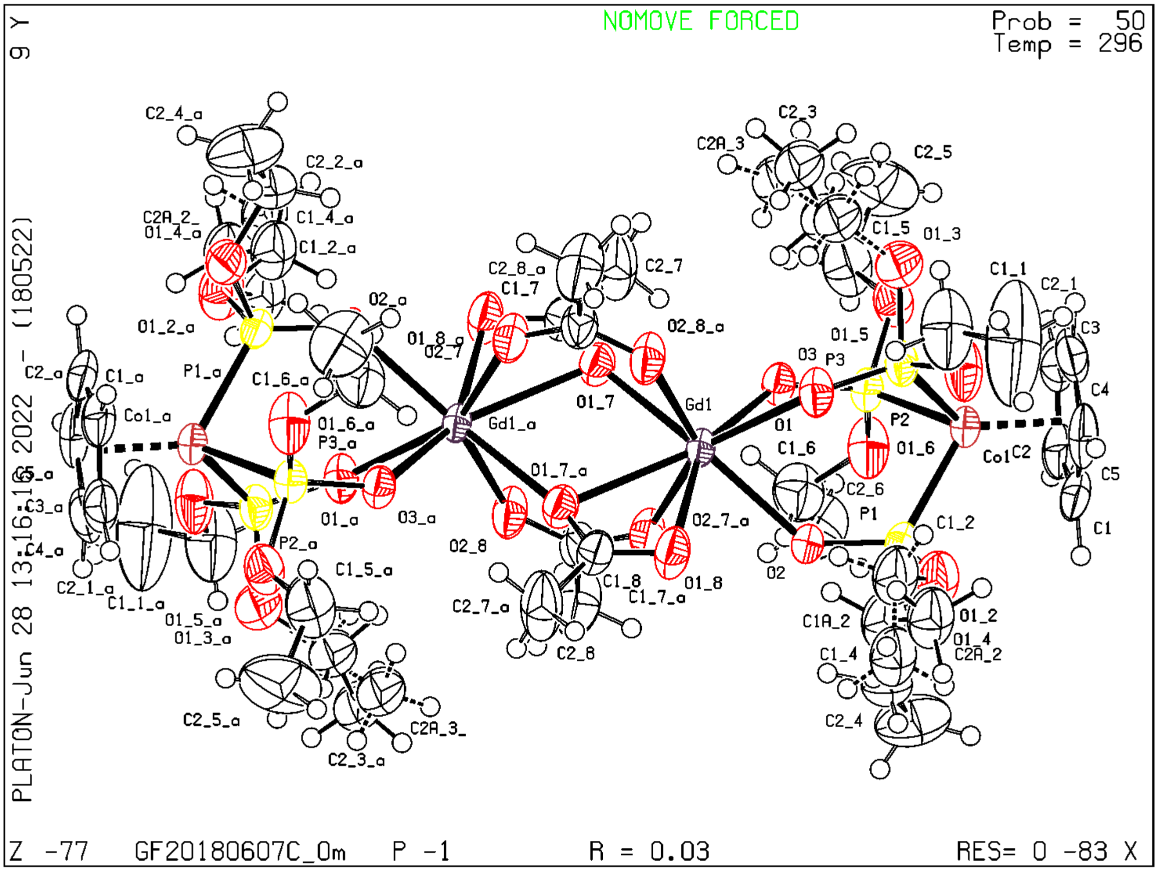

Supplement: Supplementary file 1 [file DataSheet1.PDF]
