# Supplementary material for: Syntheses, structures, and magnetic properties of acetate-bridged lanthanide complexes based on a tripodal oxygen ligand
Source: Front Chem. 2022 Sep 19;10:1021358. doi: 10.3389/fchem.2022.1021358 (PMC9527289; doi:10.3389/fchem.2022.1021358)
Supplement: Supplementary file 2 [file DataSheet2.docx]

Supplementary Material for

Syntheses, Structures and Magnetic Properties of Acetate-Bridged Lanthanide Complexes Based on a Tripodal Oxygen Ligand

Yu Sheng^1^, Yu-Jing Jiang^1^, Zi-Hang Cheng^1^, Ru-Chan Liu^1^, Jing-Yuan Ge^2*^, and Feng Gao^1*^

^1^School of Chemistry & Materials Science, Jiangsu Normal University, Xuzhou 221116, P. R. China

^2^College of Chemistry and Materials Engineering, Wenzhou University, Wenzhou 325035, P. R. China

.**Contents**

**Materials and characterization methods**

**X-ray Crystallography**

**Supplementary Table S1.** Crystallographic data of all complexes.

**Supplementary Table S2.** Selected bond lengths (Å) and bond angles (°) for all complexes**.**

**Supplementary Table S3**. Shape analysis for the study of central lanthanide ions coordination sphere (The *S* values indicate the proximity to the ideal polyhedron, *S* = 0 corresponds to the non-distorted polyhedron).

**Supplementary Figure S1.** UV-Vis absorption spectra for all complexes in CH_2_Cl_2_ (*c* = 3 × 10^−5^ M).

**Supplementary Figure S2.** Molecular structure diagram of **2** (Tb green; Co aqua; O red; P pink; and C grey). The H atoms are omitted for clarity.

**Supplementary Figure S3.** Molecular structure diagram of **3** (Ho green; Co aqua; O red; P pink; and C grey). The H atoms are omitted for clarity.

**Supplementary Figure S4.** Molecular structure diagram of **4** (Gd green; Co aqua; O red; P pink; and C grey). The H atoms are omitted for clarity.

**Supplementary Figure S5.** Temperature-dependent in-phase (χ') and out-of-phase (χ'') ac susceptibilities in the frequency of 999 Hz for **1** under *H_dc_* = 0 Oe.

**Supplementary Figure S6.** Temperature-dependent in-phase (χ') and out-of-phase (χ'') ac susceptibilities in the frequency of 999 Hz for **2** under *H_dc_* = 0 Oe (left) and *H_dc_* = 2500 Oe (right), respectively.

**Supplementary Figure S7.** Temperature-dependent in-phase (χ') and out-of-phase (χ'') ac susceptibilities in the frequency of 999 Hz for **3** under *H_dc_* = 0 Oe (left) and *H_dc_* = 2500 Oe (right), respectively.

**Supplementary References**

**Materials and characterization methods**

Lanthanide acetate hydrate and solvents were directly purchased and used without additional purification. The tripodal ligand NaL_OEt_ were prepared according to a reported literature [S1]. Infrared spectra (IR) spectra data between 400 and 4000 cm^−1^ were obtained on a Bruker TENSOR 27 spectrophotometer using KBr tablet method. Elemental analyses for C and H elements were performed using an Elementar Vario MICRO analyzer. UV-Vis spectra were recorded with a UV-3600 spectrophotometer. Powder X-ray diffraction (PXRD) measurements were carried out through a Bruker D8 Advance X-ray (Cu *Kα*, λ = 1.5418 Å) diffractometer. Magnetic properties data were measured on a Quantum Design MPMS-SQUID-VSM magnetometer. Diamagnetic corrections were applied for the sample holder and calculated using Pascal’s constants [S2]

**X**-**ray** **Crystallography**

X-Ray single crystal data for all complexes were collected at 296(2) K through a Bruker SMART Apex II CCD diffractometer (Mo *Kα*, λ = 0.71073 Å). Crystal structures were solved by direct methods using *SHELXT* [S3] and refined by full-matrix least-squares based on *F^2^* using the *SHELXL* program [S4]. *SADABS* program were applied for multi-scan absorption [S5]. H atoms were laid in geometrically idealized positions with isotropic refinement using riding mode and non-H atoms based on anisotropic displacement parameters were refined.

**Supplementary Table S1.** Crystallographic data of all complexes.

|  | **1** | **2** | **3** | **4** |
| --- | --- | --- | --- | --- |
| formula | C_42_H_82_Co_2_Dy_2_O_26_P_6_ | C_42_H_82_Co_2_Tb_2_O_26_P_6_ | C_42_H_82_Co_2_Ho_2_O_26_P_6_ | C_42_H_82_Co_2_Gd_2_O_26_P_6_ |
| formula weight | 1631.75 | 1624.59 | 1636.61 | 1621.25 |
| crystal system | triclinic | Triclinic | Triclinic | Triclinic |
| space group | *Pī* | *Pī* | *Pī* | *Pī* |
| *a* / Å | 9.8444(10) | 9.8434(6) | 9.8488(9) | 9.8497(7) |
| *b* / Å | 10.1916(10) | 10.1878(7) | 10.1850(9) | 10.1837(8) |
| *c* / Å | 17.0109(17) | 17.0374(11) | 16.9611(15) | 17.0879(13) |
| *α* / deg | 92.7490(10) | 92.8070(10) | 92.7350(10) | 92.8080(10) |
| *β* / deg | 97.0580(10) | 96.9900(10) | 97.1280(10) | 96.9850(10) |
| *γ* / deg | 106.3080(10) | 106.2680(10) | 106.4070(10) | 106.2280(10) |
| *V* / Å^3^ | 1619.5(3) | 1621.76(18) | 1613.3(2) | 1627.3(2) |
| Z | 1 | 1 | 1 | 1 |
| *ρ_calcd_* /g cm^−3^ | 1.673 | 1.663 | 1.684 | 1.654 |
| *μ* / mm^−1^ | 3.005 | 2.877 | 3.152 | 2.732 |
| *θ* / deg | 2.505−26.022 | 2.546−26.021 | 2.642−26.371 | 2.321−26.369 |
| F(000) | 818 | 816 | 820 | 814 |
| GOF (*F^2^*) | 1.044 | 1.044 | 1.009 | 0.984 |
| R_1_, wR_2_ (I>2σ(I)) | 0.0271, 0.0691 | 0.0272, 0.0700 | 0.0318, 0.0785 | 0.0293, 0.0750 |
| R_1_, wR_2_ (all data)  (all data) | 0.0305, 0.0713 | 0.0305, 0.0725 | 0.0371, 0.0817 | 0.0339, 0.0785 |
| CCDC number | 2182586 | 2182587 | 2182588 | 2182589 |

**Supplementary Table S2.** Selected bond lengths (Å) and angles (°) for all complexes.

|  | **1** | **2** | **3** | **4** |
| --- | --- | --- | --- | --- |
| Bond Distances (Å) | | | | |
| Ln1−O1 | 2.338(2) | 2.352(2) | 2.325(3) | 2.365(2) |
| Ln1−O2 | 2.311(2) | 2.321(2) | 2.298(3) | 2.334(2) |
| Ln1−O3 | 2.332(2) | 2.342(2) | 2.315(3) | 2.356(2) |
| Ln1−O1_7 | 2.337(2) | 2.349(2) | 2.315(3) | 2.362(2) |
| Ln1−O1_8 | 2.345(3) | 2.357(2) | 2.327(3) | 2.366(3) |
| Ln1−O1_7#1 | 2.537(2) | 2.542(2) | 2.530(3) | 2.554(2) |
| Ln1−O2_7#1 | 2.439(3) | 2.455(3) | 2.423(3) | 2.467(3) |
| Ln1−O2_8#1 | 2.354(3) | 2.363(3) | 2.343(3) | 2.380(3) |
| Bond Angles (°) | | | | |
| O1−Ln1−O2 | 75.70(9) | 75.61(9) | 75.91(10) | 75.34(9) |
| O1−Ln1−O3 | 77.58(9) | 77.29(9) | 77.64(10) | 76.92(9) |
| O2−Ln1−O3 | 78.08(9) | 77.88(9) | 78.34(10) | 77.70(9) |
| O1­_8−Ln1− O1_7 | 76.44(9) | 76.23(9) | 76.54(11) | 76.16(10) |
| O1­_7−Ln1− O1_7#1 | 73.18(9) | 73.17(9) | 72.97(11) | 73.31(10) |
| O1­_7#1−Ln1− O2_7#1 | 52.01(8) | 51.68(8) | 52.29(9) | 51.48(8) |
| O1­_7−Ln1− O2_8#1 | 73.01(9) | 72.97(9) | 73.16(11) | 72.97(10) |
| Ln1-O1_7-Ln1#1 | 106.82(9) | 106.83(9) | 107.02(11) | 106.69(9) |

Symmetry code: #1 -x+1,-y+1,-z+1.

**Supplementary Table S3**. Shape analysis for the study of central lanthanide ions coordination sphere (The S values indicate the proximity to the ideal polyhedron, S = 0 corresponds to the non-distorted polyhedron).

|  | **1-*S*_Dy_** | **2-*S*_Tb_** | **3-*S*_Ho_** | **4-*S*_Gd_** |
| --- | --- | --- | --- | --- |
| Cube (*O*_h_) | 10.780 | 10.779 | 10.761 | 10.857 |
| Square antiprism (*D*_4d_) | 1.619 | 1.642 | 1.608 | 1.695 |
| Triangular dodecahedron (*D*_2d_) | 2.278 | 2.333 | 2.261 | 2.336 |

**
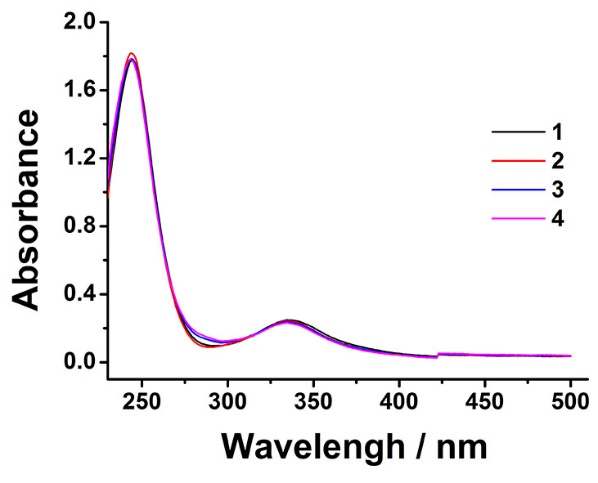
**

**Supplementary Figure S1.** UV-Vis absorption spectra for all complexes in CH_2_Cl_2_ (*c* = 3 × 10^−5^ M).


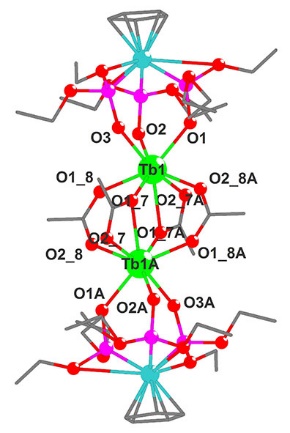


**Supplementary Figure S2.** Molecular structure diagram of **2** (Tb green; Co aqua; O red; P pink; and C grey). The H atoms are omitted for clarity.

~~
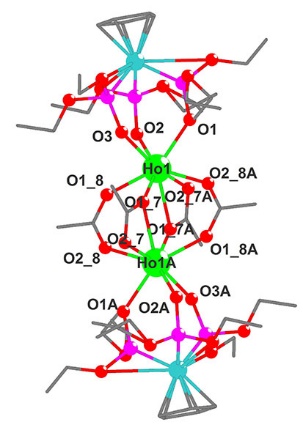
~~

**Supplementary Figure S3.** Molecular structure diagram of **3** (Ho green; Co aqua; O red; P pink; and C grey). The H atoms are omitted for clarity.

**
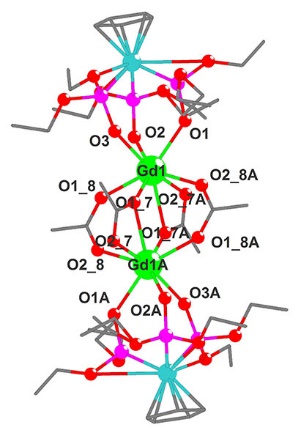
**

**Supplementary Figure S4.** Molecular structure diagram of **4** (Gd green; Co aqua; O red; P pink; and C grey). The H atoms are omitted for clarity.


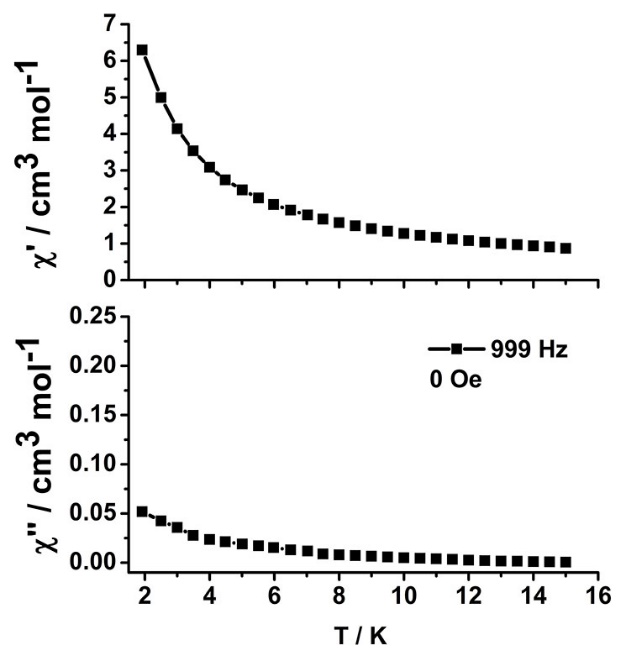


**Supplementary Figure S5.** Temperature-dependent in-phase (χ') and out-of-phase (χ'') ac susceptibilities in the frequency of 999 Hz for **1** under *H_dc_* = 0 Oe.


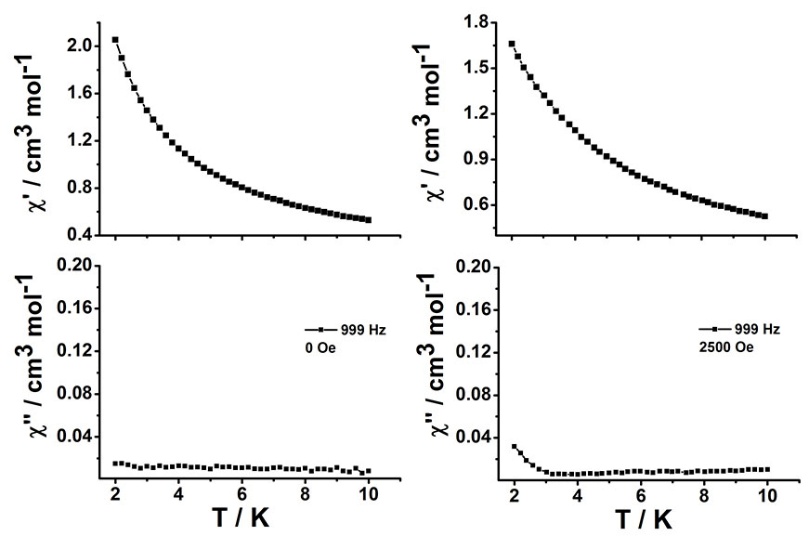


**Supplementary Figure S6.** Temperature-dependent in-phase (χ') and out-of-phase (χ'') ac susceptibilities in the frequency of 999 Hz for **2** under *H_dc_* = 0 Oe (left) and *H_dc_* = 2500 Oe (right), respectively.


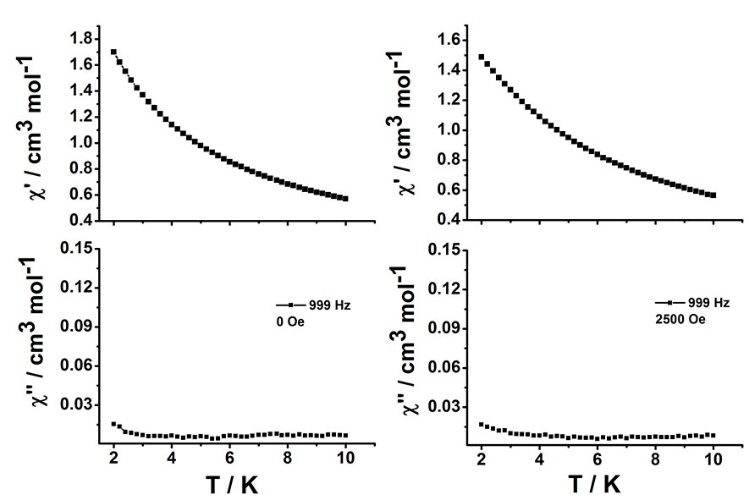


**Supplementary Figure S7.** Temperature-dependent in-phase (χ') and out-of-phase (χ'') ac susceptibilities in the frequency of 999 Hz for **3** under *H_dc_* = 0 Oe (left) and *H_dc_* = 2500 Oe (right), respectively.

**Supplementary References**

S1 Kläui, W., Müller, A., Eberspach, W., Boese, R., and Goldberg, I. (1987). Crystal structure and coordination chemistry of the pentane-soluble sodium salt of an oxygen tripod ligand. *J*. *Am*. *Chem*. *Soc*. 109, 164−169.

S2 Boudreaux, E. A., and Mulay, L. N. (1976). Theory and Application of Molecular Paramagnetism; John Wiley & Sons: New York, 491-494.

S3 Sheldrick, G. M. (2015). SHELXT - Integrated space-group and crystal-structure determination. *Acta Crystallogr., Sect. A: Fundam. Crystallogr*. 71, 3-8.

S4 Sheldrick, G. M. (2015). Crystal structure refinement with SHELXL. *Acta Crystallogr., Sect. C: Struct. Chem.* 71, 3-8.

S5 Sheldrick, G. M. (1996). *SADABS an empirical absorption correction program;* Bruker Analytical X-ray Systems: Madison, WI.
